# Assessing the Groundwater Quality at a Saudi Arabian Agricultural Site and the Occurrence of Opportunistic Pathogens on Irrigated Food Produce

**DOI:** 10.3390/ijerph121012391

**Published:** 2015-10-05

**Authors:** Dhafer Alsalah, Nada Al-Jassim, Kenda Timraz, Pei-Ying Hong

**Affiliations:** Water Desalination and Reuse Center, Division of Biological and Environmental Science and Engineering, King Abdullah University of Science and Technology (KAUST), Thuwal 23955-6900, Saudi Arabia; E-Mails: Dhafer.salah@kaust.edu.sa (D.A.); nada.aljassim@kaust.edu.sa (N.A.-J.); kenda.timraz@kaust.edu.sa (K.T.)

**Keywords:** groundwater, food safety, total nitrogen, coliforms, quantitative microbial risk assessment

## Abstract

This study examines the groundwater quality in wells situated near agricultural fields in Saudi Arabia. Fruits (e.g., tomato and green pepper) irrigated with groundwater were also assessed for the occurrence of opportunistic pathogens to determine if food safety was compromised by the groundwater. The amount of total nitrogen in most of the groundwater samples exceeded the 15 mg/L permissible limit for agricultural irrigation. Fecal coliforms in densities > 12 MPN/100 mL were detected in three of the groundwater wells that were in close proximity to a chicken farm. These findings, coupled with qPCR-based fecal source tracking, show that groundwater in wells D and E, which were nearest to the chicken farm, had compromised quality. Anthropogenic contamination resulted in a shift in the predominant bacterial phyla within the groundwater microbial communities. For example, there was an elevated presence of *Proteobacteria* and *Cyanobacteria* in wells D and E but a lower overall microbial richness in the groundwater perturbed by anthropogenic contamination. In the remaining wells, the genus *Acinetobacter* was detected at high relative abundance ranging from 1.5% to 48% of the total groundwater microbial community. However, culture-based analysis did not recover any antibiotic-resistant bacteria or opportunistic pathogens from these groundwater samples. In contrast, opportunistic pathogenic *Enterococcus faecalis* and *Pseudomonas aeruginosa* were isolated from the fruits irrigated with the groundwater from wells B and F. Although the groundwater was compromised, quantitative microbial risk assessment suggests that the annual risk incurred from accidental consumption of *E. faecalis* on these fruits was within the acceptable limit of 10^−4^. However, the annual risk arising from *P. aeruginosa* was 9.55 × 10^−4^, slightly above the acceptable limit. Our findings highlight that the groundwater quality at this agricultural site in western Saudi Arabia is not pristine and that better agricultural management practices are needed alongside groundwater treatment strategies to improve food safety.

## 1. Introduction

The production of food is intricately linked to water consumption, and agricultural production remains the thirstiest sector relative to domestic needs and industrial use. 70%–80% of the freshwater supplies are withdrawn in Saudi Arabia to feed the demand for water by the agriculture sector [1,2]. While each person drinks on average 2 to 4 L of water per day, up to 2000 to 5000 L of water are consumed to produce the three meals each person takes in per day [3,4]. In many water-stressed countries like Saudi Arabia, the production of food is conducted at an unsustainable rate. Groundwater supplies are pumped up at alarming rates and stored in wells for agricultural irrigation. The heavy exploitation of non-renewable groundwater supplies has rendered aquifers particularly vulnerable to contamination.

Use of groundwater with compromised quality can impact the food quality during the pre-harvest stage. According to the Ministry of Health in Saudi Arabia, the number of reported foodborne illnesses has steadily been increasing for more than a decade [5]. In a questionnaire-based survey conducted in 2009, about 14.9% of the 1064 surveyed schoolchildren in Jeddah reported suffering from diarrhea [6]. While a portion of the foodborne diarrhea in Saudi Arabia arises due to poor hygienic practices, some of these cases may also be due to consumption of fresh produce compromised by contaminated irrigation water. The likelihood of this cannot be overlooked as viral and bacterial pathogens have been found in irrigation water of other countries [7,8]. Similarly, microcystins from cyanobacterial bloom were also detected in groundwater wells of Saudi Arabia, and they were found to accumulate in vegetables irrigated with water from those contaminated wells [9]. This exemplifies the need to survey the groundwater quality for possible contamination events and to perform systematic microbial risk assessment on the food produce.

Currently, there is no existing database that collates information relating to the groundwater quality in Saudi Arabia. In a number of studies that have been conducted in the region, groundwater samples were found to contain nitrate contents exceeding the 15 mg/L level recommended by the local regulating agencies [10,11,12,13]. Furthermore, fecal coliforms were present in 21.4% of the well waters that were tested throughout Saudi Arabia over a one-year period in 1989 [10]. Although these studies provide insights to the groundwater quality in Saudi Arabia, many date back to almost two decades ago, and the groundwater quality may have changed since then.

There is a need for monitoring efforts on the groundwater quality to be renewed, and for focusing on elucidating the potential impact of compromised groundwater quality on food safety. Such information is especially crucial because the Kingdom is moving towards using treated wastewater as one of the national water taps for agricultural irrigation [14]. To reuse the treated wastewater, various strategies are under consideration, including recharging the dry riverbeds or injecting the treated wastewater into underlying aquifers prior to pumping them for use in agricultural irrigation [15]. There is a need to establish the current groundwater quality and compare it to the treated wastewater quality to determine if there would be any significant risk in using the latter for recharging of groundwater aquifers.

This study serves to address the knowledge gaps relating to the quality of groundwater in Saudi Arabia and the quality of fruits irrigated with the groundwater. To achieve these aims, conventional methods to determine the nutrient content and coliform numbers were used and complemented with molecular-based approaches. These approaches include quantitative PCR (qPCR)-based microbial source tracking and high-throughput sequencing to determine the relative abundance of genera associated with opportunistic pathogens. Bacterial isolates were cultivated from both groundwater and fruits, and phylogenetically identified based on the 16S rRNA genes. Lastly, foodborne pathogens that were isolated and identified were assessed for their extent of risk imposed on public consumers upon ingestion through quantitative microbial risk assessment (QMRA).

## 2. Materials and Methods

### 2.1. Sampling Site and Sampling Procedure

**Figure 1:**
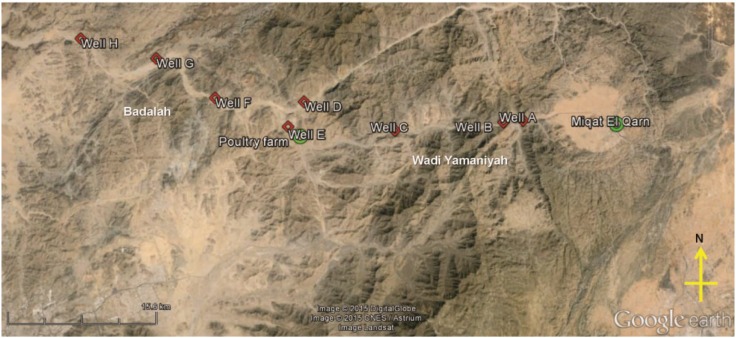
Sampling site in Taif, Saudi Arabia. Groundwater samples were collected from wells A through H. Groundwater direction flows from A to H. Tomatoes and green peppers irrigated with groundwater from wells B and F were sampled. Green circles denote potential sources of anthropogenic contamination. Miaqt E1 Qarn is a gathering venue for pilgrims.

The study area is located in a narrow valley north of the city of Mecca, Saudi Arabia, with approximate GPS coordinates of 21.7° N, 40° E. Groundwater flows from the northwest direction from an area called Wadi Yamaniyah to Badalah. Two sampling trips were made, on 12 and 26 March 2014, to collect groundwater samples from eight different wells (Figure 1). For the first sampling trip, groundwater was collected from three wells, A, B and C, in Wadi Yamaniyah. All three wells are located downstream of a pilgrimage gathering venue called Miqat El Qarn (21.6° N, 40.4° E). For the second sampling trip, groundwater from wells A, B and C, as well as an additional five wells D through H were sampled downstream from a poultry farm in Badalah. Wells D and E were located approximately 6 and 1 km downstream, respectively, from the poultry farm. These groundwater samples can be categorized into two groups based on their proximity to the poultry farm. Group 1 comprised samples from wells A, B and H that were located > 20 km from the poultry farm. Group 2 comprised samples from wells C through G that were located < 20 km from the poultry farm. To collect groundwater samples, mechanical pumps were activated to pump up groundwater from a depth of approximately 0.3 km below the ground surface. The water was flushed for approximately 3 min prior to collection into sterile polyethylene bottles. A total of 5 L of groundwater was collected from each well. In addition, approximately 1 kg of both tomatoes and green peppers were sampled at sites that utilized groundwater from wells B and F for irrigation. All samples were placed into a cooler and kept cool during transport. Once at the laboratory, samples were stored at 4 °C for 1–2 days prior to analyses and preparation as detailed in subsequent Section 2.2 through Section 2.3.

### 2.2. Measurement of Nutrient Content, Coliforms and Numbers of Total Cells in Groundwater

Groundwater samples were analyzed for their non-purgable organic carbon (NPOC), total nitrogen (TN), and numbers of total cells and coliforms. Water samples were first filtered through 0.45-µm Whatman^TM^ Puradisc 25-mm syringe filters (GE Healthcare, Little Chalfont, Buckinghamshire, UK) and diluted 8-fold prior to measurement using the TOC Analyzer Wet Oxidation/NDIR Method Model (Shimadzu Scientific Instruments, Japan). Deionized water and internal standards of known total TN and NPOC concentrations were used as negative and positive controls, respectively, and were measured along with the water samples. Deionized water had an average NPOC content of 0.80 ± 0.08 mg/L and was lower than that detected in the groundwater samples. Total and fecal coliform numbers were determined by the standard EPA method number 9221 [16]. Growth media were prepared from commercially available dry mixes, namely, Fluka lauryl sulfate and brilliant green bile lactose broth (Sigma-Aldrich, Buchs, Switzerland) were used for total coliforms; and Fluka EC broth (Sigma-Aldrich, Buchs, Switzerland) was used for fecal coliforms. A set of growth media with no groundwater inoculation was also incubated as control blanks. All control blanks were absent of coliforms. The total cell numbers of bacteria in the groundwater samples were determined by the Accuri C6 flow cytometer (BD Biosciences, San Jose, CA) based on protocols described previously [17].

### 2.3. Groundwater Filtration and DNA Extraction

Water samples (2 L) were filtered through 0.45-µm Whatman Nuclepore^TM^ track-etched polycarbonate membrane filters (GE Healthcare Life Sciences, Little Chalfont, UK). The membranes were cut into thin strips with sterile razor blades, and microbial genomic DNA was extracted using the UltraClean^®^ Soil DNA Isolation Kit (MoBio, Carlsbad, CA, USA) with minor adjustments applied to the protocol [18]. All DNA concentrations were quantified using an Invitrogen Qubit^®^ 2.0 fluorometer (Thermo Fisher Scientific, Carlsbad, CA, USA).

### 2.4. Quantitative PCR (qPCR)

qPCR primer assays that target the 16S rRNA genes of human-associated *Bacteroides* spp. (*i.e.*, *B. vulgatus*, *B. fragilis* and *B. uniformis*) [17,19] and the inorganic ion metabolism gene of *B. fragilis* unique to chicken feces [20] were used for fecal source tracking. All primer sequences used in this study are listed in Supplementary Table S1. The qPCR standards were prepared from the respective gene amplicons from *B. vulgatus* BCRC12903, *B. uniformis* JCM5828, *B. fragilis* BCRC10619, as well as from the CP2-9 amplicon product from genomic DNA of chicken feces. The gene amplicons were cloned into either the pGEM-T easy vector (Promega, US) or Invitrogen pCR^TM^4-TOPO^®^ TA vector (Thermo Fisher Scientific, Carlsbad, CA, USA), and transformed into One Shot ® TOP10 *E. coli* competent cells. Plasmids were sequenced for the gene inserts to ensure perfectly matched sequences to the primer assays, and qPCR reactions were then performed with these plasmids as standards based on procedures described previously [17]. The amplification factors of the standards ranged from 1.59 to 1.96, with R-squared values of 0.95–0.99. The copy numbers of each tested gene in the samples were determined by fitting the standard curve. All no-template controls (NTCs) of Bufm1018, Bfrg1024 and Bvg1016 were undetermined and were ignored if the threshold cycle (Cq) for the lowest detectable concentration of the target gene in a sample was > 40. All NTCs of CP2-9 were negative with C_q_ values > 32 and were ignored if the C_q_ for the lowest detectable concentration of target genes in a sample was 28. All NTCs of reference 16S rRNA genes were negative with C_q_ values > 35 and were ignored if the C_q_ for the lowest detectable concentration of target gene in a sample was 31.

### 2.5. 16S rRNA Gene Amplicon-based High-throughput Sequencing and Analysis

High-throughput sequencing reactions were performed to provide information on the total microbial community [17]. The controls for the PCR reactions were negative for amplification. The 16S rRNA gene amplicons obtained were ~400 bp and were then purified using the Wizard^®^ Genomic DNA Purification Kit (Promega, Madison, WI, USA). The purified products were sent to Korea Macrogen for sequencing on Ion Torrent PGM 314 chips. Raw sequence reads from Ion Torrent PGM sequencing were handled in similar procedure as that described earlier [17]. Microbial richness for each sample was obtained from the rarefaction curves based on a defined sequencing depth of 3250 sequences (Supplementary Figure S1). To annotate the 16S rRNA gene sequences obtained from high-throughput sequencing, RDP Classifier was used for taxonomical assignments at a 95% confidence level [21]. After annotation, the relative abundances of the individual bacterial and archaeal genera were calculated, collated and then square-root transformed. The transformed dataset was then computed for their Bray-Curtis similarities and represented graphically for spatial distribution in a multidimensional scaling (mMDS) plot using Primer-E version 7 [22]. The environmental data collated from TN, NPOC, coliform counts, flow cytometry and qPCR were also compiled and inputted into Primer-E version 7 to generate a principal component analysis (PCA) plot. The BEST (Bio-Env) procedure was performed on Primer-E version 7 to match biotic patterns from high-throughput sequencing to environmental patterns [23]. This will generate the best environmental variable that correlates with the biotic pattern on the mMDS plot.

### 2.6. Isolation and Phylogenetic Identification of Bacterial Isolates Based on 16S rRNA Genes

The skins of the tomatoes and peppers were peeled to collect 50 g from each type of fruit. Then, each 50-g sample of peels was individually blended with 250 mL of deionized water to approximate typical household food preparation conditions. A total of 50 mL of the blend was then mixed with 50 mL of Miller LB broth (Sigma-Aldrich, Buchs, Switzerland) and incubated for 24 h at 37 °C to enrich the bacterial community. After incubation, the enrichment was left to stand for approximately 15 min, and a portion of the supernatant was aliquoted for 10^4^, 10^6^, and 10^8^ dilution in 1 × PBS. Each dilution (100 µL) was then spread on media plates for further incubation at 37 °C for 48–65 h. The media plates included MacConkey (Sigma-Aldrich, Buchs, Switzerland) and Brilliant Green (HiMedia Laboratories, Pennsylvania, US) agar, and both media types either with or without antibiotics. Two different types of antibiotics, 8 µg/mL meropenem (Sigma-Aldrich, Buchs, Switzerland) and 8 µg/mL ceftazidime (Sigma-Aldrich, Buchs, Switzerland), were used to test for the presence of antibiotic-resistant bacteria (ARB) in the fruit blends. Meropenem was used because it is a carbapenem within the new class of beta-lactam antibiotics. Carbapenem is typically used as a last line of defense for the treatment of many gram-negative bacterial infections [24]. Therefore, bacterial pathogens resistant to carbapenem may result in increased mortality or morbidity upon host infection. Ceftazidime is a third-generation cephalosporin and bacterial isolates resistant to ceftazidime typically encode for extended-spectrum beta-lactamase. Opportunistic bacterial pathogens isolated from meropenem- and ceftazidime-supplemented agar plates are hence likely to be those associated with nosocomial infections [25].

The approximate number of bacterial colonies per 50 g of peels was calculated based on:

(1) $$\text{CFU/50 g}=\frac{\left(\text{Dilution factor})(\text{Plate count}\right)}{\text{0.1 mL}}\times \frac{\text{100 mL of LB mixture}}{\text{50 mL of fruit blend}}\times \underset{\text{blend}}{250\text{mL of fruit}}$$

Colonies growing on the antibiotics-supplemented media plates were randomly selected and re-streaked twice to acquire pure bacterial cultures from the peels.

Similarly, bacterial isolates present in the groundwater samples were obtained from wells B and F by first adding 5 mL of the groundwater samples to 25 mL of Miller LB broth (Sigma-Aldrich, Buchs, Switzerland). The mixture was incubated for 24 h at 37 °C prior to spreading on the MacConkey and Brilliant Green agar plates. Colonies growing on the media plates were randomly selected and re-streaked twice to acquire pure cultures. All colonies were then extracted for DNA by the heat-lysis method or using the DNeasy Blood and Tissue Kit (Qiagen, Hilden, Germany) based on the manufacturer’s protocol. The 16S rRNA genes of bacterial isolates were amplified and sent in for Sanger sequencing at the KAUST Genomics core lab [17]. The sequencing results were BLASTN (Basic Local Alignment Tool) and searched against the National Center for Biotechnology Information (NCBI) 16S rRNA genes database.

### 2.7. Antibiotic Susceptibility of Pseudomonas Aeruginosa

*P. aeruginosa* isolates were further tested for their minimum inhibitory concentrations against a range of antibiotics. This is because *P. aeruginosa* is an opportunistic pathogen that was determined in this study to be present at abundances higher than the recommended risk of 10^−4^. The minimum inhibitory concentrations (MIC) of antibiotics tested for *P. aeruginosa* were 256, 128, 64, 32, 16, 8, 4, 2 and 0 µg/mL each of ampicillin, kanamycin, gentamicin, erythromycin, trimethoprim, sulfamethoxazole, tetracycline, ciprofloxacin or ceftazidime in Miller LB broth (Sigma-Aldrich, Buchs, Switzerland). MIC tests were prepared in a 96-well plate format by inoculating 4 µL of an overnight culture of the bacterium into individual wells containing 200 µL of the respective Miller LB broth with and without antibiotics. The 96-well plates containing the inoculants were then grown overnight at 37 °C and the optical density at wavelength 600 nm (OD_600_) was subsequently measured using the Spectromax 340pc microplate spectrophotometer (Molecular Devices, Sunnyvale, CA, USA). The threshold value to determine the status of a bacterial isolate as resistant to the antibiotic was set at > 70% of the OD_600nm_ value obtained in the absence of antibiotics, which ranged from an OD_600nm_ value of 0.5 to 0.8.

### 2.8. Quantitative Microbial Risk Assessment (QMRA)

The microbial risks arising from the presence of *E. faecalis* and *P. aeruginosa* in the fruits were further evaluated by QMRA. Phylogenetic identification of isolates denoted the presence of *E. faecalis* and *P. aeruginosa* at an individual fraction of 0.40 and 0.075, respectively, relative to the average plate counts of viable antibiotic-resistant isolates obtained from the antibiotic-supplemented plates. At a 95% confidence interval, the median concentration of *E. faecalis* and *P. aeruginosa* in the respective fruits were obtained by multiplying the total number of antibiotic-resistant bacteria with their corresponding individual fraction. An assumed 2.0 × 10^−6^ probability of transmission of opportunistic pathogenic species from water to the fruit surfaces was used [26]. The main exposure route considered for QMRA was through ingesting the fruit based on an assumption of 70 kg average body weight per person and that each individual consumed an average 2.9 g/kg/d [27] of a mixture of tomatoes and peppers. In addition, it was assumed that 10% of the consumed mass was derived from the fruit peels. The risk from the opportunistic pathogenic species was characterized using the exponential distribution model for daily risks:

(2) $$\text{P }(\text{response})=1-{\text{e}}^{\left(-\text{k}\times \text{dose}\right)}$$

where k is a numerical constant that denotes the probability of an organism to survive to reach and infect a host, and P is the probability of infection or death. The k constant is 2.19 × 10^−11^ for *E. faecalis* [28] and 1.87 × 10^−8^ for *P. aeruginosa* [29], obtained from dose-response studies of both bacterium administered to hosts through the gastrointestinal route or through blood injection.

Annual risk was calculated based on the equation below:

(3) $${\text{P}}_{\text{annual}}=1-{\left(1-{\text{P}}_{\text{daily}}\right)}^{\text{number of exposure days per year}}$$

The number of exposure days per year was assumed to be 365 days. Compared with the beta-poisson models, exponential models tend to be more sensitive to low dose concentrations of opportunistic pathogens, and would result in an exponential increment of risks at low doses. The combination of a year-round exposure and exponential model would thus reflect a worst-case scenario analysis of the associated risks. There is currently no legislation or guideline to denote the permissible level of risk incurred from consuming fruits and vegetables. To provide perspectives on the microbial risk outcomes, the annual risk evaluated in this study was compared against a microbial risk of 10^−4^. This risk level of 10^−4^ was proposed to be the permissible level of risk for drinking water standards in the Netherlands [30].

### 2.9. Nucleotide Sequence Accession Numbers

All high-throughput sequencing files were deposited in the Short Read Archive (SRA) of the European Nucleotide Archive (ENA) under study accession number PRJEB9501. All Sanger-based sequences of the 16S rRNA genes of *E. faecalis* and *P. aeruginosa* mentioned in Section 2.8 are listed in Supplementary Table S2.

## 3. Results

### 3.1. Chemical and Microbial Quality of Groundwater Samples

For all of the 11 groundwater samples collected, chemical (*i.e.*, total nitrogen, TN and non-purgable organic carbon, NPOC) and microbial (*i.e.*, total cell counts, total coliforms and fecal coliforms) parameters indicative of the groundwater quality were measured. The TN and NPOC of the wells water samples ranged between 15.2–61.3 mg/L and 10.6–70.6 mg/L, respectively (Table 1). The TN concentrations in the water samples collected from wells < 20 km distance from the chicken farm were significantly higher than those collected from wells > 20 km from the chicken farm (One-way ANOVA, F = 29.47, *p* = 0.00). The NPOC was, however, not significantly different between the two groups of samples (One-way ANOVA, F = 0.02, *p*-value = 0.89). Total coliforms at an abundance of > 1600 MPN/100 mL were detected only in groundwater samples collected from wells D, E and F but not from the remaining wells. Fecal coliforms were detected in wells E and F at an abundance of 1600 and 920 MPN/100 mL, respectively (Table 1). Well D had a detectable but lower density of 12 MPN/100 mL of fecal coliforms in its groundwater. The 16S rRNA gene copies determined by qPCR were highest in groundwater sampled from wells D and E, and were 33-fold higher than the average 16S rRNA gene copies determined in the other groundwater wells (Table 1).

**Table 1:** Chemical and microbial quality of groundwater samples. A (I), B (I) and C (I) denote groundwater sampled from well A, B and C on the first sampling trip. A (II), B (II) and C (II) denote groundwater sampled from well A, B and C on the second sampling trip.

Well Name	Group Number	Total Nitrogen, TN	Non-Particulate Organic Carbon, NPOC	Total Coliforms	Fecal Coliforms	16S rRNA Gene Copies
		Average (mg/L) ± Standard Deviation		MPN/100 mL		Copies/L ± Standard Deviation
A (I)	1	19.6 ± 0.2	69.1 ± 2.0	None detected		1.66 × 10^9^ ± 2.52 × 10^8^
B (I)	1	23.0 ± 0.1	16.1 ± 0.2			1.97 × 10^7^ ± 5.87 × 10^5^
C (I)	2	53.7 ± 0.3	70.6 ± 2.8			5.19 × 10^7^ ± 4.96 × 10^6^
A (II)	1	23.1 ± 0.6	33.6 ± 0.3			5.46 × 10^7^ ± 1.25 × 10^6^
B (II)	1	37.6 ± 1.0	34.9 ± 14.2			2.32 × 10^7^ ± 9.14 × 10^5^
C (II)	2	61.3 ± 5.6	22.7 ± 2.2			3.94 × 10^7^ ± 3.63 × 10^6^
D	2	55.1 ± 2.9	10.6 ± 0.9	>1600	12	1.28 × 10^10^ ± 2.56 × 10^8^
E	2	40.8 ± 0.5	12.1 ± 0.01	>1600	1600	1.14 × 10^9^ ± 5.94 × 10^8^
F	2	49.5 ± 0.6	68.8 ± 0.5	>1600	920	4.53 × 10^7^ ± 2.65 × 10^5^
G	2	42.2 ± 2.2	69.9 ± 2.9	None detected		2.95 × 10^7^ ± 2.49 × 10^5^
H	1	15.2 ± 0.1	70.6 ± 0.3			3.14 × 10^6^ ± 5.78 × 10^5^

### 3.2. qPCR-based Fecal Source Tracking

qPCR-based fecal source tracking was performed to determine if the groundwater samples were contaminated by either human or chicken fecal discharges. None of the groundwater samples were positive for the chicken-specific bacterial marker. Human fecal contamination was positively determined when two or more human-associated *Bacteroides* spp. were present. Sporadic occurrence of human fecal contamination was observed for the groundwater obtained from wells A through C across the two sampling trips. Only groundwater from well A was positive for *B. vulgatus* and *B. uniformis* during the first sampling trip, with each *Bacteroides* sp. present at an abundance of 2.31 × 10^5^ ± 3.55 × 10^4^ and 1.14 × 10^5^ ± 8.27 × 10^3^ copies/L, respectively. However, neither *Bacteroides* spp. was detected in well A on the second sampling trip. Instead, *B. fragilis* and *B. vulgatus* were present in wells B and C at an abundance ranging from 68.6 to 1.49 × 10^5^ copies/L. Well D was positive for all three human-associated *Bacteroides* with an abundance ranging from 4.68 × 10^3^ to 2.22 × 10^5^ copies/L, while well E was present for *B. fragilis* and *B. uniformis* at an abundance of 2.00 × 10^3^ ± 5.36 × 10^2^ and 1.48 × 10^4^ ± 1.96 × 10^3^ copies/L, respectively. None of the wells F through H were positive for human-associated *Bacteroides* spp.

### 3.3. Multivariate Analysis of Microbial Communities on a Metric Multidimensional Scaling Plot

**Figure 2:**
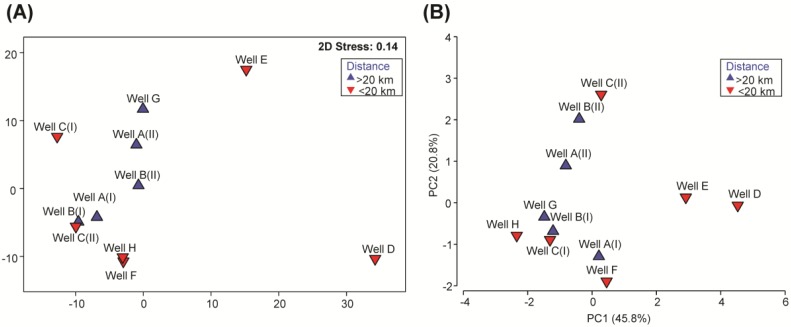
Clustering ordination analyses of groundwater wells A through H. (**A**) threshold metric dimensional scaling plot of groundwater microbial communities, (**B**) principal component analysis of water quality data. Data collated include the abundance of human-associated *Bacteroides*, 16S rRNA gene copy number, total cells, coliform density, total nitrogen and total organic carbon content.

The relative abundances of specific bacterial genera and unclassified bacterial groups detected in the different groundwater samples were analyzed on a multidimensional scaling plot, MDS (Figure 2A). Multivariate analysis revealed that microbial communities in the groundwater from wells D and E were distinctly different, and shared an average 32.1% and 43.5% similarity, respectively, with the other groundwater samples (Figure 2A). At the phylum level, *Proteobacteria* accounted for the most predominant phylum in all groundwater samples, with relative abundances ranging from 32.7% to 97.7% of the total microbial community (Supplementary Figure S2). Unlike the other groundwater samples, which were also comprised of predominant phyla *Bacteroidetes*, *Actinobacteria*, *Firmicutes* and *Deinococcus-Thermus* and unclassified Bacteria, groundwater obtained from well D was mainly comprised of *Proteobacteria* (97.7%) and *Bacteroidetes* (1.6%). In addition, groundwater in wells A, E and G had an elevated presence of phylum *Cyanobacteria* (Supplementary Figure S2) compared with the remaining wells. The microbial richness of the groundwater in wells D and E identified at a sequencing depth of 3250 sequences was 296 and 540 OTUs, respectively (Supplementary Figure S1), and was up to 4.8-fold lower than that detected in the other wells.

### 3.4. Multivariate Analysis of Water Quality Parameters and Correlation to the Microbial Pattern

The water quality parameters that were measured in this study, *i.e*., TN, NPOC, total coliforms, fecal coliforms, total cell counts, copy numbers of 16S rRNA genes and human-associated *Bacteroides* spp., were collated and presented on a principle component analysis (PCA) plot (Figure 2B). Multivariate analysis on the water quality parameters revealed that the water quality in the groundwater from wells D and E was distinctly different from the remaining wells, and clustered apart from the other samples along principle component axis 1 (PC1). This distribution of samples along PC1 was mainly due to the high abundance of human-associated *B. fragilis*, *B. uniformis* and coliforms in wells D and E compared with the levels detected in the other wells. The high TN content in all groundwater samples resulted in the presence of ammonia-oxidizing and nitrite-oxidizing microorganisms. Specifically, the main ammonia-oxidizing microorganisms detected were *Nitrososphaera* and *Nitrosopumilus*, at an average relative abundance of 0.08% ± 0.08% and 0.34% ± 0.47%, respectively, across all groundwater samples. Nitrite-oxidizing microorganisms were mainly comprised of *Nitrospira*, which was ubiquitously present in all groundwater samples at an average relative abundance of 0.19% ± 0.13%. To further determine which water quality parameter correlated with the clustering differences of the microbial communities in groundwater as shown in Figure 2A, the resemblance matrix of the microbial data was matched against the water quality patterns exhibited on the PCA plot by the Bio-Env (BEST) procedure. The fecal coliform count exhibited the best correlation to the clustering pattern of microbial communities at a 90% confidence interval (*ρ* = 0.585, *p* = 0.076), and likely explained the apparent dissimilarities of microbial communities in wells D and E compared with other wells.

### 3.5. Molecular-based Detection of Genera Associated with Opportunistic Pathogens and Bacterial Isolation from Groundwater Samples

**Figure 3:**
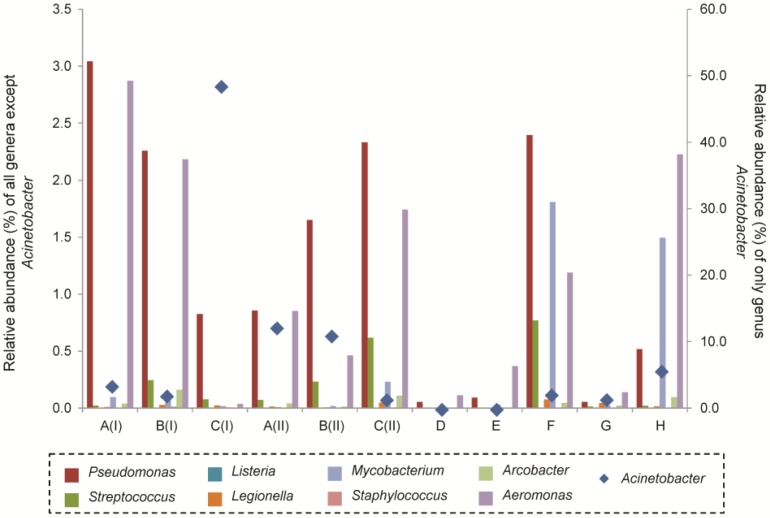
Relative abundance of genera associated with opportunistic pathogens in the groundwater collected from respective wells A through H. The relative abundance was compared to the total groundwater microbial community analyzed using high-throughput sequencing.

Based on high-throughput sequencing of the total 16S rRNA genes, the genus *Pseudomonas* accounted for a relative abundance of 2.4% in well F and 2.0% in well B, both of which were wells that supplied groundwater for irrigation (Figure 3). In addition, high-throughput sequencing also detected the presence of other genera associated with opportunistic pathogens (e.g., *Streptococcus*, *Legionella*, *Mycobacterium*, *Staphylococcus*, *Aeromonas*) at lower relative abundance of up to 2.0% of the total microbial community. However, genus *Acinetobacter* was detected at high relative abundance of up to 48.6% in the total microbial community of groundwater from well C sampled during the first sampling trip (Figure 3). The relative abundance of *Acinetobacter* in the nearby wells A and B also accounted for 11.1%–12.3% of total microbial community (Figure 3). To further examine the bacterial species associated with these genera, a further enrichment of the bacterial community was performed. Estimated bacterial counts on both MacConkey and brilliant green bile lactose agar media without any antibiotics were 9.0 × 10^4^ and 3.3 × 10^4^ CFU/mL, respectively, in groundwater from well F. The estimated bacterial counts in well B on both types of media were 5.3 × 10^3^ and 1.6 × 10^4^ CFU/mL of groundwater. No antibiotic-resistant bacteria were recovered from either groundwater sample. Phylogenetic identification based on the 16S rRNA genes showed that three main bacterial species were isolated from well F. Among the 26 bacterial isolates recovered from well F, 7.7% was identified as *Acidovorax temperans*, 46.2% was identified as *Pseudomonas putida* and 46.2% as *Pseudomonas anguilliseptica* at 99% 16S rRNA gene similarity. In contrast, *Rhizobium endophyticum*, *R. galegae*, and *R. paknamense* were detected at 6.7%, 40.0% and 3.3%, respectively, of the total bacterial isolates recovered from well B (n = 30). The remaining bacterial isolates were mainly identified as *Pseudomonas peli* (46.7%) and *Staphylococcus epidermidis* (3.3%).

### 3.6. Bacterial Isolation in Irrigated Fruits

An average 2.08 × 10^11^ and 3.20 × 10^11^ CFU from 50 g of fruit peels were obtained on antibiotic-free MacConkey agar and antibiotic-free brilliant green bile lactose agar plates, respectively. The number of ceftazidime-resistant bacteria was higher than meropenem-resistant bacteria for both types of fruits. An average 1.64 × 10^10^ CFU/50 g of tomato peels were obtained on ceftazidime-supplemented media. This is 4-log higher than the average 1.25 × 10^6^ CFU/50 g of tomato peels that were counted on the meropenem-supplemented media. In addition, an average of 9.76 × 10^9^ CFU/50 g of green pepper peels were counted on the ceftazidime-supplemented plate but no meropenem-resistant bacteria were counted. A total of 17 colonies obtained from the fruits harvested near well B were sequenced for their 16S rRNA genes and were determined to be *Enterococcus faecalis* (n = 16) and *Vibrio natriegens* (n = 1). A total of 23 colonies obtained from the fruits harvested near well F were determined to be *Enterococcus mundtii* (n = 13), *Pseudomonas monteilii* (n = 3), *Pseudomonas entomophila* (n = 4) and *Pseudomonas aeruginosa* (n = 3).

### 3.7. Microbial Risk Associated with Ingestion of Tomatoes and Peppers Harvested from Sampling Sites

A quantitative microbial risk assessment (QMRA) was performed for *Enterococcus faecalis* and *P. aeruginosa*, both of which are potential opportunistic pathogens isolated from the fruits that were irrigated with groundwater from wells B and F. Phylogenetic identification of isolates denoted that *E. faecalis* and *P. aeruginosa* were present at a proportion of 0.40 and 0.075, respectively, relative to the total counts of antibiotic-resistant isolates. The median exposure dose for *E. faecalis* and *P. aeruginosa* through ingestion was 7.47 × 10^2^ cells/d and 1.40 × 10^2^ cells/d, respectively. Consequently, for a worst case scenario, the annual risk arising from ingestion of *E. faecalis* in the fruits was 5.98 × 10^−6^, and from ingestion of *P. aeruginosa* in fruits was 9.55 × 10^−4^ (Table 2). Since *P. aeruginosa* is an opportunistic pathogen and the annual risk is slightly above the benchmarked risk of 10^-4^, these isolates were further tested for their minimum inhibitory concentrations of the respective antibiotics. All of the *P. aeruginosa* were highly resistant to trimethoprim, ampicillin, sulfamethaxazole, kanamycin and erythromycin, but were susceptible or only able to withstand low concentrations (<4 µg/mL) of gentamicin, tetracycline, ciprofloxacin and ceftazidime (Supplementary Table S3).

**Table 2:** Elaboration on the quantitative microbial risk assessment (QMRA) for *Pseudomonas aeruginosa* and *Enterococcus faecalis*, and the assumptions made to facilitate QMRA.

Parameters	Annotation	Assumed Value	Reference
Average weight per person (kg)	A	70	[27]
Total amount of fruits consumed (g/kg/d, assuming that it is an equal portion of only tomatoes and green pepper)	B	2.9	
Proportion of consumed fruits amounting from the peels	C	0.10	
Transmission probability of bacterium from water to fruit surfaces	D	2.00 × 10^−6^	[26]
Median cell numbers of genus *Pseudomonas* per 50 g of peels over 95% confidence interval	E	1.73 × 10^8^	
Median cell numbers of genus *Enterococcus* per 50 g of peels over 95% confidence interval	F	9.20 × 10^8^	
Exposure dose of *P. aeruginosa* (cells/event) = A **×** B **×** C **×** D **×** E / 50 g	1.40 × 10^2^		
k of *P. aeruginosa* (Exponential model; derived from the LD50 dose of *P. aeruginosa* required for infections via murine gastrointestinal tract)	1.87 × 10^−8^		[29]
Point estimate of risk arising from *P. aeruginosa* = 1 − exp (−k **×** exposure dose)	2.62 × 10^−6^		
Annual risk arising from *P. aeruginosa* = 1 − (1-point estimate)^365 days per year	9.55 × 10^−4^		
Exposure dose of *E. faecalis* (cells/event) = A **×** B **×** C **×** D **×** F / 50 g	7.47 × 10^2^		
k of *E. faecalis* (Exponential model; derived from LD50 dose of *E. faecalis* required for peritonitis via blood injection)	2.19 × 10^−11^		[28]
Point estimate of risk arising from *E. faecalis* = 1 − exp (−k **×** exposure dose)	1.64 × 10^−8^		
Annual risk arising from *E. faecalis* = 1 − (1-point estimate)^365 days per year	5.98 × 10^−6^		

## 4. Discussion

Over the past two decades, non-renewable supplies of groundwater have been extensively used in Saudi Arabia for agriculture irrigation. Large abstraction of non-renewable groundwater supplies has led to falling groundwater levels and rendered aquifers to become more vulnerable to either saline intrusion or infiltration of surface water from the nearby environment [31,32]. In Saudi Arabia, groundwater used for unrestricted irrigation is required to meet various standards including but not limited to < 2.2 CFU/100 mL, 15 mg/L in total nitrogen and 40 mg/L in total organic carbon [33]. However, regular monitoring of the groundwater quality is seldom conducted and little is known about the groundwater quality in Saudi Arabia. Our findings revealed that except for groundwater from well H, TN in the groundwater samples was higher than the 15 mg/L total nitrogen recommended by the local standards (Table 1).

Well H, which had a TN content at a permissible level of 15.2 mg/L, was located farthest away from all possible fecal contamination sources identified in this study. It is likely that the high TN content in the groundwater of wells A through F may be due to the use of fertilizers to increase soil productivity, and/or anthropogenic contamination from the nearby vicinities (*i.e*., pilgrimage gathering venue and poultry farm). Agricultural soils in Saudi Arabia, including the soils at the agriculture farms studied here, are generally sandy and impoverished. The localized soil and sand are also highly permeable and porous, and nutrients can infiltrate easily into the vadose zones of groundwater aquifers [2,12,13]. Although the farmers at the studied sites adopt summer fallow, they remain reliant on heavy usage of fertilizers and pesticides to achieve a good crop yield. It is estimated that approximately 300 kg of vegetative-animal compost was applied as fertilizer per year at the agricultural farms in this study. Further compounding this problem, only 40% of Saudi Arabia’s land mass is connected by sewage pipelines [34]. Human settlement areas, particularly those around agricultural farms, are not served by sanitary networks and have to rely on local septic tanks to store their municipal wastes. However, septic tanks are designed to discharge supernatant effluent contaminated with human sewage into the surrounding area. Due to the rampant use of septic tanks in many areas of Saudi Arabia, it was estimated that effluent discharged from the septic tanks caused the groundwater level to rise by up to 0.41 m between 1996 and 2000 [35]. Clearly, septic tanks are a major source of anthropogenic contamination in Saudi Arabia and may account for the sporadic detection of human-associated *Bacteroides* spp. and fecal coliforms in groundwater wells A through E.

Similar to a previous study that monitored the groundwater quality in the Midwest USA [36], specific bacterial populations in the groundwater correlated with the geochemistry of the groundwater. In this study, the high TN content selected for the ubiquitous presence of nitrifying microbial populations, specifically ammonia-oxidizing archaea (AOA) *Nitrososphaera* and *Nitrosopumilus* and nitrite-oxidizing microorganisms *Nitrospira*, in the groundwater samples. AOA was in higher abundance than the bacterial counterpart (AOB), and is in agreement with previous studies that detected the ubiquitous presence of AOA in environments, including the ocean, drinking water distribution systems and soils [37,38,39]. In all of these environments where they were detected, AOA played a dominant role in oxidizing ammonia to nitrite, and nitrite is further utilized by nitrite-oxidizing bacteria as part of the nitrogen cycle.

In addition to the correlation between TN and the nitrifying bacterial communities, the abundance of fecal coliforms was further determined through the BEST analysis to exhibit a relatively higher correlation with the microbial patterns in comparison to the other measured parameters (e.g., TN, TOC, 16S rRNA gene copies and cell counts). This correlation suggests that fecal contamination can perturb the groundwater microbiota. For example, wells D and E had an abundance of fecal coliforms exceeding regulations and a corresponding distinct shift in the bacterial phyla *Proteobacteria* and *Cyanobacteria* was observed in the groundwater. More specifically, wells D and E exhibited a notably high relative abundance of *Proteobacteria* but had a microbial richness that was 4.8-fold lower than that in other groundwater samples. A previous study also noted increased relative abundance of *Proteobacteria* and a lower microbial richness in the more impacted groundwater wells [36]. Furthermore, a meta-analysis of marine habitats identified 30%–50% reduction in species richness in habitats exposed to contamination events [40]. It is likely that anthropogenic contamination events have detrimentally impacted the indigenous microbial communities by removing rare and/or keystone species from the ecosystem, in turn reducing the resilience of communities to other stressors [41].

Despite a positive detection of fecal coliforms and a high TN concentration in the groundwater downstream of the poultry livestock production farm, qPCR-based source tracking did not detect chicken-specific markers in any of the groundwater samples. The lack of detection of chicken-specific markers in the groundwater downstream of the chicken farms (e.g., wells D and E) may be due to the low abundance of *Bacteroides fragilis* in animals compared with humans [42,43], which resulted in a lower detection sensitivity of such animal-associated fecal markers in the environment. In addition, amplification factors of the host-specific primer assays were below 2, and these values were similar to those reported by other studies that used the same primer assays for qPCR-based fecal source tracking [17,19]. Clearly, better primer assays should be developed to amplify the target genes more effectively. However, owing to the technical difficulties in identifying 16S rRNA gene regions that are unique to and representative of host-specific bacterial targets, other quantitative molecular-based approaches that can circumvent the qPCR limitations can be used in future studies to more effectively trace fecal contamination events arising from poultry production farms [44].

Despite the low amplification efficiency of the qPCR assays, human-associated *Bacteroides* were detected in wells D and E as well as sporadically in wells A through C. These observations were in agreement with the fecal coliform counts in wells D and E. However, the lack of detection of fecal coliforms in wells A to C by culture-based methods was not in line with the sporadic positive detection of human-associated *Bacteroides* in these wells by molecular methods. This may be due to the inability to resuscitate and grow viable but non-culturable coliforms that were stressed or injured in the environmental water matrix [45]. In addition, DNA detected by molecular-based qPCR approaches has a longer persistence than the viable cells [45,46]. This in turn enabled detection of the gene markers, but a positive detection may not be representative of a recent contamination event.

High-throughput sequencing further revealed that the genus *Acinetobacter* was present in high relative abundance in the groundwater in wells A through C during both sampling periods (Figure 3). Compared with the relative abundance of 1.2% for the genus *Acinetobacter* in raw wastewater collected from a Saudi Arabian wastewater treatment plant [18], the relative abundances of the genus *Acinetobacter* in these wells were much higher. In Saudi Arabia, *Acinetobacter baumannii* is one of the most common antibiotic-resistant bacteria isolated from clinical samples [47,48]. It remains unknown if *A. baumannii* is equally prevalent in the non-nosocomial environment in Saudi Arabia and whether it would act as a major causative pathogenic agent in non-nosocomial settings. The short read lengths obtained in this study did not allow identification at the species level. Cultivation-based analysis of the groundwater in well B did not reveal the presence of any *A. baumannii*, likely because some *Acinetobacter* species do not grow well on MacConkey agar [49] and were not optimally enriched for growth on a coliform-selective media like the brilliant green bile lactose agar under the incubation conditions of 37 °C for 48–65 h. Alternatively, PCR amplification may be biased towards the relative abundance of *Acinetobacter* spp. To illustrate, out of the 32 *Acinetobacter* genomes available in the rrnDB database, the *Acinetobacter* genus has on average 6 copies of 16S rRNA genes per genome [50]. The copy number of 16S rRNA genes per genome among *Acinetobacter* spp. is higher than the mean copy number of 4 among the 2635 microbial genomes currently available and analyzed in the same rrnDB database [50]. As a result of the relatively higher number of rrn copy numbers in *Acinetobacter*, molecular methods that involve the use of gene amplification may have presented a quantitative bias towards this genus with higher copy numbers [51,52], and hence result in a higher abundance of *Acinetobacter* spp. shown in high-throughput sequencing datasets than the actual cell numbers within the sample.

The majority of the bacterial species isolated were *Pseudomonas* species including *P. putida* and *P. peli*, which are commonly found in soil and water (*i.e*., of environmental origins). None of the isolated *Pseudomonas* was a human opportunistic pathogen except for *P. anguilliseptica*, which is a known fish pathogen [53] and may be of potential concern to nearby aquaculture farms. Therefore, despite the groundwater showing a higher relative abundance of the genus *Acinetobacter* and *Pseudomonas* compared with the treated wastewater effluent sampled from a wastewater treatment plant in Saudi Arabia [18], there were no significant health concerns arising from the use of the groundwater. Furthermore, unlike a previous study, which recovered antibiotic-resistant *Aeromonas* spp. and *Pseudomonas* spp. from the treated wastewater effluent [18], no meropenem-resistant or ceftazidime-resistant bacteria were recovered from the groundwater in well B and F.

However, fruits harvested from the agricultural farms in Wadi Yamaniyah and Badalah had a high abundance of ARB. This observation is in agreement with another local Saudi Arabia case study, which found that approximately 2.3% of the detected bacterial isolates in raw vegetables produced extended spectrum beta-lactamase, therefore conferring resistance to a wide spectrum of beta-lactam antibiotics [54]. In this study, *E. faecalis* and *P. aeruginosa* account for the two predominant ARBs that were enriched from the fruit peels. Given that *E. faecalis* and *P. aeruginosa* were not isolated from the nearby groundwater, it is likely that the occurrence of these two opportunistic pathogenic species on the fruits arose due to agricultural management practices (e.g., application of fertilizers in close contact with the fruits and crops) or from other above-surface sources at each individual farm. A separate study noted that antibiotic-resistant bacteria are ubiquitous on vegetables and that antibiotic resistance genes possibly predate any anthropogenic effects [55]. Hence, it would only be of concern to public health if the viable bacteria remaining on the fruits are foodborne pathogens that will result in an associated risks higher than the acceptable probability of 10^−4^ per annum [30]. To address this concern, the associated risks from ingesting tomatoes or peppers from the site were further explored through QMRA. The annual risk arising from ingestion of *E. faecalis* in the fruit peels was 5.68 × 10^−6^ and this risk is within the acceptable probability. However, in a worst-case scenario, the annual risk incurred from accidental ingestion of *P. aeruginosa* on fruit peels was 9.55 × 10^−4^ and slightly higher than the acceptable probability (Table 2).

To the best of our knowledge, there are no reports of any foodborne disease outbreak associated with *P. aeruginosa* in the region within the past few years at the time of conducting this study. It is possible that the QMRA may have overestimated the risks associated with *P. aeruginosa* due to several limitations of our QMRA approach. Firstly, the assumed consumption rate of 365 days per year and at 0.29 g/kg/d of both types of fruit peels may not be representative of the actual consumption rates among the Saudi Arabian population. A questionnaire-based survey should be conducted in the future to better assess the consumption rates in the examined population and hence provide a more accurate QMRA. Secondly, the exposure doses of *E. faecalis* and *P. aeruginosa* were obtained from enrichment cultures and are semi-quantitative. The cell culture numbers may be inadvertently biased towards bacterial species that can be recovered easily on the nutrient media. Examples of other opportunistic pathogenic species that may be potentially present in the fruit peels include *Acinetobacter baumannii*. However, these species cannot be assessed for their risks due to the inability to recover these microorganisms. Future studies should aim to monitor *Acinetobacter* spp., particularly those that are opportunistic pathogenic species, through a combination of molecular methods and cultivation using the appropriate medium (e.g., CHROMagar^®^, Leeds *Acinetobacter* base agar). Thirdly, the QMRA performed in this study is a single point estimate, and variability and an uncertainty analysis were not included. Such variability and uncertainty may be incurred when assuming the probability of transmission of bacteria from water to the surface of the fruits. To illustrate, Gerba and Choi estimated that the transmission probability ranged from 0.00021% to 9.4% [26], and may hence account for a broad variability and uncertainty in the estimated microbial risks. In addition, the use of exponential models and the assumption of a daily consumption of these fruits when performing QMRA represent a worst-case scenario analysis. Thus, risk values are only indicative and the actual ingestion risks arising from *P. aeruginosa* may be low despite being slightly above 10^−4^. For future work, these uncertainties in QMRA should be better addressed through a more comprehensive monitoring effort that will analyze a larger dataset throughout the year to account for seasonal differences in the groundwater and food quality.

## 5. Conclusions

This study provided baseline information on the groundwater quality at an agricultural site situated in the western part of Saudi Arabia. It was found that groundwater sampled at this site was contaminated with a high nutrient content and with human fecal sewage. The presence of fecal coliforms arising from anthropogenic contamination perturbed the groundwater microbial community and lowered the microbial richness. Molecular-based analysis further reported a much higher relative abundance of the genus *Acinetobacter* compared with treated wastewater evaluated in an earlier study using the same approach. Although culture-based analysis did not recover any opportunistic human pathogenic species like *A. baumannii* and *P. aeruginosa*, *P. anguilliseptica,* which is a fish pathogen, was particularly abundant in the groundwater of well F. The harvested fruits irrigated with the groundwater showed a high abundance of ARB even though ARB was not detected in the groundwater samples. Examples of ARB that could be isolated from the food produce included *E. faecalis* and *P. aeruginosa*. The annual risk arising from the consumption of *E. faecalis* was lower than 10^−4^, and not likely to present a significant public health concern. However, in a worst-case scenario, the annual risk arising from *P. aeruginosa* was 9.55 × 10^−4^ and slightly above the acceptable probability of 10^−4^. Our findings highlighted that the groundwater quality at this agricultural site in western Saudi Arabia was not pristine and that better agricultural management practices are needed alongside groundwater treatment strategies to improve food safety.

## Supplementary Files

Supplementary File 1
